# Detection of SARS-CoV-2 omicron variants by immunochromatographic kit

**DOI:** 10.1016/j.heliyon.2023.e20913

**Published:** 2023-10-12

**Authors:** Satoshi Oshiro, Fuminori Mizukoshi, Naeko Mizutani, Makoto Akiwa, Jun-Ichiro Sekiguchi, Tatsuya Tada, Yuki Yamamoto, Yoko Tabe, Takashi Miida, Teruo Kirikae

**Affiliations:** a Department of Microbiology, Juntendo University School of Medicine, Tokyo, Japan; b Tochigi Prefectural Institute of Public Health and Environmental Science, Utsunomiya, Tochigi, Japan; c Microbiology Research Division, Kohjin Bio Co., Ltd., Saitama, Japan; d Department of Clinical Laboratory Medicine, Juntendo University School of Medicine, Tokyo, Japan

**Keywords:** Immunochromatographic kit, Severe acute respiratory syndrome coronavirus 2 (SARS-CoV-2) omicron variants

## Abstract

An immunochromatographic kit using antibodies against recombinant N protein of an omicron B.1.1.529 of severe acute respiratory syndrome coronavirus 2 (SARS-CoV-2) was developed to detect SARS-CoV-2 omicron variants. The kit detected omicron variants (BA.1.18, BA.1.1, BA.2, BA.2.12.1, BA.2.75, BA.4.1, BA.4.6, BE.1, BA.5.2.1, XE, BF.7, BF.7.4.1, XBB.1, XBB.1.5 and BQ.1.1) as well as Wuhan strain and a delta variant.

## 1 Introduction Text

Severe acute respiratory syndrome coronavirus 2 (SARS-CoV-2) resulted from the coronavirus disease 2019 (COVID-19) pandemic which emerged in December 2019 (https://www.who.int/emergencies/diseases/novel-coronavirus-2019) [1]. During the pandemic, SARS-CoV-2 rapidly accumulated genetic mutations, including alpha, delta, gamma and omicron variants (https://www.who.int/activities/tracking-SARS-CoV-2-variants) [2]. As of November 2022, among SARS-CoV-2 sequences shared through Global Initiative on Sharing Avian Influenza Data (GISAID), 99.7% were those of the omicron variant (https://www.who.int/publications/m/item/weekly-epidemiological-update-on-covid-19---26-october-2022) [3].

We developed two immunochromatographic assay (ICA) kits to detect SARS-CoV-2 Wuhan strain[4] and a delta variant of SARS-CoV-2 [5], respectively. These kits used monoclonal antibodies against the nucleocapsid (N) protein [amino acid (aa) 1–419] of SARS-CoV-2 Wuhan strain. The present study describes an improved ICA kit to detect SARS-CoV-2 omicron variants.

**Fig. 1:**
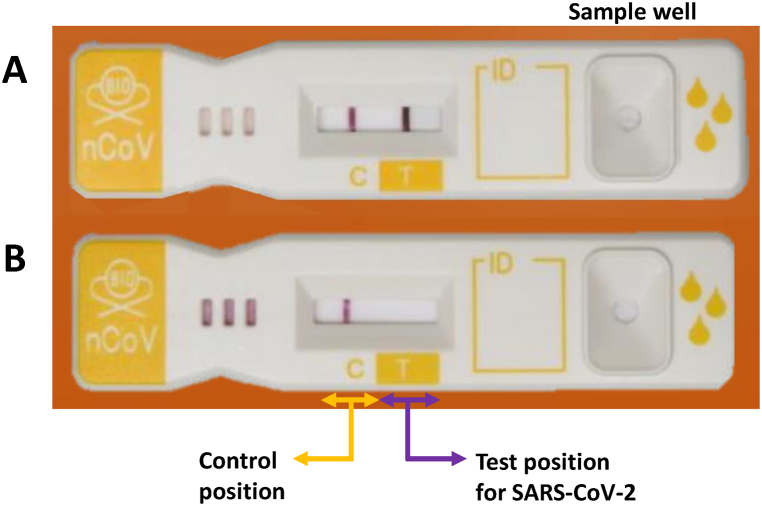
Details of the immunochromatographic assay kit (KBM LineCheck nCoV (device-type). Samples showing two lines, one each at the control and test position for SARS-CoV-2 were positive for SARS-CoV-2 (A). Samples showing a single line at the control position were negative for SARS-CoV-2 (B).

To prepare monoclonal antibodies, rats were immunized with recombinant SARS-CoV-2 N protein from omicron B.1.1.529 variant (hCoV-19/Japan/TY38-873/2021). The monoclonal antibodies were purified using Spin Column Based Antibody Purification kits (Cosmo Bio USA, Carlsbad, CA, USA) from the culture supernatants of antibody-producing hybridomas. Antibody N3J13 was found to be the most effective capture antibody and N3J15 the most effective detection antibody in sandwich ELISA tests against the recombinant N protein of SARS-CoV-2 omicron B.1.1.529, and recognized aa 1–220 and 210–419 regions, respectively (data not shown). The ICA kit [KBM Linecheck nCoV (device-type)] was developed by coating nitrocellulose membranes with a capture monoclonal antibody against SARS-CoV-2 (N3J13) and with a goat anti-rat IgG antibody as described[4]. A detection monoclonal antibody against SARS-CoV-2 (N3J15) was conjugated with colloidal gold nanoparticles and immersed into glass fiber. A nasopharyngeal swab sample (50 μl) obtained from a COVID-19 patient was added to 200 μl of Tris-based buffer (pH 7.6), of which 90 μl (three droplets) was added to a sample well of the ICA (Fig. 1 A and B). Line formation on the strip was assessed within 15 min after immersing it.

Due to the presence of human-derived enzymes and variability in quality, nasopharyngeal swabs may be unreliable samples to determine the lower detection limit of an ICA kit. In contrast, virus culture supernatants are more reliable. To determine the lower detection limit of the ICA, culture supernatants of SARS-CoV-2 omicron variants, including BA.1.18 [hCoV-19/Japan/TY38-873/2021, 4.2 × 10^5^ tissue culture infectious dose 50 (TCID50)/mL], BA.1.1 (hCoV-19/Japan/TY38-871/2021, 1.0 × 10^6^ TCID50/mL), BA.2 (hCoV-19/Japan/TY40-385/2022, 1.0 × 10^7^ TCID50/mL), BA.2.12.1 (hCoV-19/Japan/TY41-721/2022), BA.2.75 (hCoV-19/Japan/TY41-716/2022, 1.0 × 10^7^ TCID50/mL), BA.4.1 (hCoV-19/Japan/TY41-703/2022, 1.0 × 10^4^ TCID50/mL), BA.4.6 (hCoV-19/Japan/TY-41-763/2022, 3.7 × 10^7^ TCID50/mL), BE.1 (hCoV-19/Japan/TY-41-702/2022, 3.2 × 10^5^ TCID50/mL), BA.5.2.1 (hCoV-19/Japan/TY41-704/2022, 1.8 × 10^4^ TCID50/mL), XE (hCoV-19/Japan/TY41-686/2022, 1.3 × 10^7^ TCID50/mL), BF.7 (hCoV-19/Japan/TY41-820/2022, 5.6 × 10^5^ TCID50/mL), BF.7.4.1 (hCoV-19/Japan/TY41-828/2022, 5.6 × 10^5^ TCID50/mL), XBB.1 (hCoV-19/Japan/TY-41-795/2022, 3.2 × 10^5^ TCID50/mL), XBB.1.5 (hCoV-19/Japan/23–018/2022, 1.2 × 10^6^ TCID50/mL) and BQ.1.1 (hCoV-19/Japan/TY-41-796/2022, 5.6 × 10^6^ TCID50/mL), SARS-CoV-2 Wuhan strain (Pango Lineage A, 2019-nCoV/Japan/TY/WK-521/2020, 1.0 × 10^7^ TCID50/mL) and delta B.1.617.2 (hCoV-19/Japan/TY-11-927/2021, 5.6 × 10^7^ TCID50/mL) were diluted in phosphate-buffered saline (PBS). An aliquot of diluted culture supernatant (10 μl) was further diluted in 240 μl of Tris-based buffer (pH 7.6), then 90 μl of the solution was used to test the ICA. The RNA in the diluted culture supernatants was extracted using QIAamp Viral RNA Mini Kit (QIAGEN, Hilden, Germany) and quantified by quantitative reverse transcription PCR (RT-qPCR) to determine the number of SARS-CoV-2 RNA copies present. The thermal cycling conditions were: 50 °C for 30 min; 95 °C for 15 min; followed by 45 cycles of 95 °C for 15 s and 60 °C for 1 min) [6]. As shown in Table 1, the ICA kit detected all the omicron variants tested with the lower detection limits of virus concentrations from 1.56 × 10^0^ to 3.91 × 10^2^ TCID50/mL, and with those of virus RNA copies from 4.12 × 10^4^ to 3.02 × 10^5^. The lower detection limit median and average were 3.04 x 104 virus RNA copies/μl and 5.63 x 104 ± 7.09 x 104 copies/μl, respectively. The ICA kit also detected SARS-CoV-2 Wuhan (Pango Lineage A, 2019-nCoV/JPN/TY/WK-521) and delta B.1.617.2 (hCoV-19/Japan/TY11-927/2021) strains with similar lower detection limits of virus concentrations as those of the omicron variants (Table 1). The Ct values of culture supernatants at the lower detection limit of SARS-CoV-2 variants was from 19.5 to 23.8, whereas those of the Wuhan strain and the delta variant were 20.6 and 26.7, respectively (Table 1).

**Table 1:** The lower detection limit of KBM LineCheck nCoV in culture supernatants of SARS-CoV-2 Whuan and variants.

SARS-CoV-2 variants^a^	(TCID50/mL)	Virus concentration in Tris-based buffer (TCID50/mL)	Virus copies in RNA extraction solution (copies/μl)	Ct values of diluted virus solution with the lower detection
omicron BA.1.18	4.2 x 105	6.66 x 101	4.38 x 104	20.4
omicron BA.1.1	1.0 x 106	7.81 x 101	2.99 x 104	21.0
omicron BA.2	1.0 x 107	3.91 x 102	4.12 x 103	23.8
omicron BA.2.12.1	ND	ND	3.83 x 104	20.6
omicron BA.2.75	1.0 x 107	3.91 x 102	3.04 x 104	20.9
omicron BA.4.1	1.0 x 104	1.56 x 100	1.98 x 104	21.5
omicron BA.4.6	3.7 x 106	2.89 x 102	5.50 x 104	20.1
omicron BE.1	3.2 x 105	1.25 x 101	2.77 x 104	21.1
omicron BA.5.2.1	1.8 x 104	4.50 x 101	3.02 x 105	21.1
omicron XE	1.3 x 107	2.54 x 102	4.37 x 103	23.7
omicron BF.7	5.6 x 105	4.38 x 101	1.15 x 105	19.5
omicron BF.7.4.1	5.6 x 105	4.38 x 101	7.10 x 104	20.2
omicron XBB.1	3.2 x 105	2.50 x 101	2.24 x 104	21.5
omicron XBB.1.5	1.2 x 106	9.38 x 101	5.54 x 104	20.5
omicron BQ.1.1	5.6 x 106	1.09 x 102	2.60 x 104	21.3
Pango Lineage A	1.0 x 107	3.91 x 102	3.87 x 104	20.6
delta B.1.617.2	5.6 x 107	5.48 x 102	6.56 x 102	26.7

a SARS-CoV-2 Wuhan strain and variants were from National Institute of Infectious Disease, Tokyo, Japan. ND: not determined.

A total of 69 nasopharyngeal swab samples were collected from 44 COVID-19 patients and 25 volunteers during March 2021 to July 2022 in Tokyo. All 44 samples from COVID-19 patients were from Juntendo University Hospital and not arbitrarily chosen, while all 25 samples from volunteers were from Nansho Hospital, Iwate, Japan. Positive diagnosis of the 44 patients was confirmed by RT-qPCR using Ampdirect 2019-nCoV detection kit according to manufacturer's protocol (SHIMADZU Corporation, Kyoto, Japan). The Ct values of the 44 samples ranged from 12.0 to 19.9 (mean 17.0 ± 2.1, median 17.3). No RT-qPCR-positive sample with Ct > 20 was obtained. SARS-CoV-2 omicron and delta variants were identified by RT-qPCR using VirSNiP SARS-CoV-2 spike T478K and VirSNiP SARS-CoV-2 spike S371L S373P according to manufacturer's protocols (TIB MolBio, Berlin, Germany).

Of the 44 samples from patients, delta variant was detected in six obtained in period Aug. 5 to 31, 2021; omicron BA.1 in six, Jan. 12 to 29, 2022; BA.2 in six, Mar. 30 to July 16, 2022, and BA.5 in six, Jul. 11 to 17, 2022 (Table S1). No variant was identified in 20 samples obtained in period Mar. 18 to Aug. 31, 2021 (Table S1). All samples from the 44 patients were ICA-positive for SARS-CoV-2, whereas all from the 25 volunteers, which were obtained Jul. 12 to Aug. 5, 2022 were ICA-negative (Table 2).

**Table 2:** Positive rates of KBM LineCheck nCoV in nasopharyngeal swabs obtained from COVID-19 patients.

SARS-CoV-2 variants	Positive/Total samples	Positive rate
RT-qPCR-positive for SARS-CoV-2^a^		
omicron BA.1	6/6	100 %
omicron BA.2	6/6	100 %
omicron BA.5	6/6	100 %
delta	6/6	100 %
unidentified^b^	20/20	100 %
RT-qPCR-negative for SARS-CoV-2^c^		
	0/25	0 %

a A total of 44 nasopharyngeal swab samples were SARS-CoV-2 RT-qPCR positive (Ct value was less than 20) (Table S1).

b The variants of the samples were not determined.

c A total of 25 nasopharyngeal swab samples were SARS-CoV-2 RT-qPCR negative.

In this study, the ICA kit effectively detected several omicron variants of SARS-CoV-2 demonstrating it is useful to diagnose COVID-19 patients infected with current prevalent strains, albeit with less sensitivity than that of RT-qPCR (Table 1). We previously developed an ICA kit to detect SARS-CoV-2 which recognized regions of N protein in Wuhan strain (aa 1–120 and aa 111–220)[4], whereas, the present kit recognized different regions of N protein in an omicron variant (aa 1–220 and aa 210–419). Both ICA kits can detect SARS-CoV-2 Wuhan strain and omicron variants (data not shown). This ICA kit has the potential to detect variants of SARS-CoV-2 in future waves of the pandemic, because the amino acid sequence of N protein is relatively conserved compared to that of the spike (S) protein; i.e. compared to the original Wuhan strain, the current most prevalent omicron variant BA.5 has eight amino acid substitutions in the N protein (Table 3) but 34 in the S (https://gisaid.org/GISAID). Assessment and improvement of the ICA kit will be continued.

**Table 3:** Nucleocapsid protein amino acid mutations of the SARS-CoV-2 omicron variants.

SARS-CoV-2 omicron variants	Mutations^a^										
BA.1.1	P13L		E31del	R32del	S33del				R203K	G204R	
BA.2	P13L		E31del	R32del	S33del				R203K	G204R	S413R
BA.2.12.1	P13L		E31del	R32del	S33del			A152S	R203K	G204R	S413R
BA.2.75	P13L		E31del	R32del	S33del				R203K	G204R	S413R
BA.4	P13L		E31del	R32del	S33del		P151S		R203K	G204R	S413R
BA.4.6	P13L		E31del	R32del	S33del		P151S		R203K	G204R	S413R
BE.1	P13L		E31del	R32del	S33del	E136D			R203K	G204R	S413R
BA.5.2.1	P13L		E31del	R32del	S33del				R203K	G204R	S413R
XE	P13L		E31del	R32del	S33del				R203K	G204R	S413R
BF.7	P13L	G30del	E31del	R32del	S33F				R203K	G204R	S413R
BF.7.4.1	P13L	G30del	E31del	R32del	S33F				R203K	G204R	S413R
XBB.1	P13L		E31del	R32del	S33del				R203K	G204R	S413R
XBB.1.5	P13L		E31del	R32del	S33del				R203K	G204R	S413R
BQ.1.1	P13L		E31del	R32del	S33del	E136D			R203K	G204R	S413R

a Nucleocapsid protein amino acid mutations in SARS-CoV-2 omicron variants were compared with SARS-CoV-2 Wuhan strain (accession no. NC_045512.2). The amino acid mutations which each omicron variants obtained were showed.

The ICA kit seems to detect SARS-CoV-2 omicron variants as well as Pango Lineage A and delta B.1.617.2 at the same sensitivity. Preliminary analysis was unable to determine the epitopes of these monoclonal antibodies using synthesized overlapped peptides with 20 amino acids of N protein (date not shown). Further detailed epitope analysis is necessary in further studies to identify suitable and stable epitopes of N protein of SARS-CoV-2 to improve ICA kits.

## Funding

This study was supported by grants from 10.13039/501100001691Japan Society for the Promotion of Science (grant number 22K16379), a grant from the 10.13039/100009619Japan Agency for Medical Research and Development (grant number20he0622015h0001) and a joint research fund from Kohjin Bio Co., Ltd.

## Ethics statement

This study was approved by the ethics committees at Juntendo University (20–036) and Kohjin Bio Co., Ltd (02608–2106). This study complied with all relevant national regulations and institutional policies. The informed consent was obtained from all participants.

## Data availability statement

Data included in article/supp. material/referenced in articles.

## CRediT authorship contribution statement

**Satoshi Oshiro:** Writing – original draft, Investigation, Funding acquisition. **Fuminori Mizukoshi:** Resources, Investigation. **Naeko Mizutani:** Investigation. **Makoto Akiwa:** Validation, Methodology, Investigation. **Jun-Ichiro Sekiguchi:** Validation, Methodology, Investigation. **Tada Tatsuya:** Conceptualization. **Yuki Yamamoto:** Resources. **Yoko Tabe:** Resources, Conceptualization. **Takashi Miida:** Conceptualization. **Teruo Kirikae:** Writing – original draft, Conceptualization.

## Declaration of competing interest

The authors declare the following financial interests/personal relationships which may be considered as potential competing interests:

MA and JS are employees of Kohjin Bio Co., Ltd.
